# A commentary on the paper: ‘Evaluation of spice and herb as phytoderived selective modulators of human retinaldehyde dehydrogenases using a simple *in vitro* method’

**DOI:** 10.1042/BSR20211522

**Published:** 2022-01-11

**Authors:** Anna Bilska-Wilkosz

**Affiliations:** Chair of Medical Biochemistry, Jagiellonian University Medical College, Kraków, Poland

**Keywords:** cancer therapy, class 2 of aldehyde dehydrogenases, retinal dehydrogenases, retinoic acid, star anise

## Abstract

It is commonly known that aldehyde dehydrogenases (ALDHs) are a promising therapeutic target in many diseases. Bui et al.—the authors of the paper I am discussing here (*Biosci Rep* (2021) **41**(5): BSR20210491  https://doi.org/10.1042/BSR20210491)—point that there is a lack of research on the use of spices and herbs as the sources of naturally occurring modulators of ALDH activity. In order to carry out this type of research, the authors prepared ethanolic extracts of 22 spices and herbs. The main objective of the study was to investigate retinaldehyde dehydrogenases (RALDHs), of which retinal is the main substrate and ALDH2, the mitochondrial isoform, having acetaldehyde as the main substrate.

The obtained results indicated that the tested extracts exhibited differential regulatory effects on RALDHs/ALDH2 and some of them showed a potential selective inhibition of the activity of RALDHs.

## Commentary

Bui et al. in their interesting paper [1] focus their attention mainly on retinaldehyde dehydrogenases (RALDHs), which are enzymes converting retinal into retinoic acid (RA). The authors emphasized the need for the search for the compounds having an influence on the activity of these enzymes because of their potential use in cancer therapy.

The retinoid family (retinoids) comprises vitamin A (retinol) and its natural derivatives such as retinaldehyde, RA and retinyl esters, as well as many synthetic derivatives [2]. Retinoids are essential in a variety of biological processes. Retinol is a 20-carbon alcohol that consists of a cyclohexenyl ring and side chain with four double bonds (all in trans configuration), hence the name all-trans-retinol. All-trans retinal is formed in a reversible oxidation reaction of retinol catalyzed by retinol dehydrogenase (EC 1.1.1.300; RDH). 11-cis-Retinal is produced from the form all-trans via complex enzymatic mechanisms. 11-cis Retinal is a component of rhodopsin, a protein found in the retina of the eye and involved in the process of vision.

All-trans-retinoic acid (atRA) is the most active, natural derivative of vitamin A. It is produced by the irreversible oxidation of all-trans retinaldehyde. This process is catalyzed by one of three retinaldehyde dehydrogenase enzymes (RALDH1, RALDH2, RALDH3). These enzymes were identified as members of the aldehyde dehydrogenase (ALDH) family of proteins.

The mammalian aldehyde dehydrogenases (ALDH; aldehyde:NAD^+^ oxidoreductase, EC 1.2.1.3) are a family of enzymes that catalyze the oxidation reactions of aldehydes which are generated via the metabolism of alcohols. The substrates for ALDH also include aldehydes derived from metabolism of biogenic amines, drugs and other xenobiotics, as well as aldehydes in the foodstuff. The oxidation of aldehydes to their corresponding acids catalyzed by ALDHs is an irreversible process. It is commonly known that ALDHs are a promising therapeutic target in many diseases. The selected modulators of enzymatic activity of enzymes from the ALDHs family and their pharmacological potential are shown in the table below (Table 1).

**Table 1:** Selected modulators of enzymatic activity of enzymes from the ALDHs family and their pharmacological potential

Compound	Mode of action	Some pharmacological (biological) properties tested or/and used	Some references
Disulfiram	Inhibition (irreversible)	Anticancer; antimicrobial	[4,5]
Nitroglycerine	Acts as a substrate for ALDH2	Anticancer	[6]
Coprine	Inhibition (irreversible)	Displays mutagenicity and gonadotoxicity	[7]
Daidzin	Inhibition (reversible)	In ancient Chinese medicine in the anticancer prevention and as the antidipsotropic agent	[7]
Ampal	Inhibition (irreversible)	Anticancer	[8]
Gossypol	Inhibition	In ancient Chinese medicine as a male contraceptive; anticancer	[7,9]
Pargyline	Inhibition (irreversible)	In the past as an antihypertensive agent; neurodegenerative diseases such as Parkinson’s and Alzheimer’s diseases	[7,10]
Citral	Inhibition (reversible)	Antidiabetic; anti-inflammatory; anticancer; antimicrobial	[11]
Alda-1	Activator	Reduce injury caused by acute myocardial infarction (MI) and ischemia/reperfusion	[4,12]
Histamine	Activator	Bone loss due to increased osteoclastogenesis	[4]
Tamoxifen	Activator	Bone protective effects, anticancer	[4,13]

Analyses of the ALDH gene superfamily conducted so far have revealed that the human genome contains 19 putatively functional genes and three pseudogenes. There are three general classes of ALDHs: class 1 (ALDH1), class 2 (ALDH2) and class 3 (ALDH3). ALDH1 contains cytosolic proteins, class 2 includes mitochondrial proteins and class 3 comprises tumor-related proteins. The inhibitor of ALDHs, disulfiram (also known as Antabuse, DSF) inhibits the activity of all ALDH isoenzymes regardless of the *K*_M_ value. DSF has been used in the treatment of alcohol dependence, as it causes ethanol intolerance due to acetaldehyde poisoning. Acetaldehyde is approximately ten-times more toxic than ethanol. The oxidation of acetaldehyde to acetic acid is catalyzed by the members of ALDH1 and ALDH2 of the ALDH family of proteins. Cytosolic ALDH1 is present in many tissues, including the brain, but the affinity of this enzyme for acetaldehyde is relatively low (*K*_M_ approx. 50 µM). Much greater affinity for acetaldehyde is shown by mitochondrial ALDH2 (*K*_M_ < 5 μM). Thus, the mitochondrial isoenzyme ALDH2 plays the major role in biotransformation of acetaldehyde.

ALDH2 gene is composed of 13 exons. Exon 12 contains a G-to-A missense mutation, which causes that glutamate (Glu) at position 504 is replaced by lysine (Lys). The Glu^504^Lys point mutation in the ALDH2 gene, common among the population of East-Asian ethnic origin (approximately 50% of the population), causes production of a low-activity form of the enzyme, with heterozygotes Glu^504^Lys (ALDH2^*^1/2) having approximately 6% of normal ALDH2 activity and homozygotes Lys^504^ (ALDH2^*^2/2) lacking its activity (rs671 polymorphism). It causes that people bearing a low-activity mutant of ALDH2 exhibit significantly lowered alcohol tolerance due to adverse effects of acetaldehyde accumulation. On the other hand, the case–control studies indicate that the deficiency of ALDH2 activity affects the risk of cardiovascular and neurodegenerative diseases. Mei et al. [3] showed that the presence of ALDH2* 2/2 (rs67) variant was related to the reduction in the risk of essential hypertension (EH). The authors even put forward the thesis that this variant of ALDH2 could be an attractive candidate for genetic therapy of EH [3].

At this point, it is worth recalling that ALDH2 plays also a significant role in the bioactivation of nitroglycerin (glyceryl trinitrate; GTN) to nitric oxide (NO) through a mechanism involving its esterase activity [14]. GTN belongs to a class of potent organic nitrates used for almost a century to treat myocardial ischemia that induces coronary vasodilation by an NO-dependent mechanism [15]. The available literature data indicate that during the reaction of hydrolysis GTN to 1,2-glyceryl dinitrate and nitrite (NO_2_^−^), ALDH2 undergoes oxidative inhibition, causing down-regulation of this enzyme which is associated with GTN tolerance [16]. Thus, in the opinion of many authors, ALDH2 activity is a useful marker of cardiovascular oxidative stress, but the concept is still under discussion [17].

Additionally, ALDH2 is involved in the oxidation of 3,4-dihydroxyphenylglycoaldehyde (DOPGAL) and 3,4-dihydroxyphenylacetaldehyde (DOPAL) in the central nervous system (CNS). DOPGAL is an intermediate product of the metabolism of the neurotransmitter noradrenaline (NA), and DOPAL is the metabolite of the major brain neurotransmitter dopamine (DA). According to the ‘catecholaldehyde hypothesis’, DOPAL, the DA metabolite produced in the monoamine oxidase (MAO)-catalyzed reaction mediates DA toxicity *in vivo*, which indicates that DOPAL plays a role in the pathogenesis of Parkinson’s disease (PD) [18]. So, it is not surprising that many authors have shown that ALDH2 polymorphisms may be associated with the risk of the most common neurodegenerative disorders that are Alzheimer’s disease (AD) and PD. In 2000, Kamino et al. indicated in the Japanese population a significant association between an increased risk of AD and the carriage of the ALDH2^*^2/2 allele [19]. In the case of PD, the available literature data are not unambiguous. Increased levels of aldehydes in the substania nigra (SN) in patients with PD are associated with the loss of gene expression of ALDH1A1 form (rather than ALDH2 form) causing the loss of enzyme protein and activity [20]. The meta-analysis based on 5315 individuals, mostly from East Asian countries, including China, Japan and Korea indicated that the polymorphism locus of ALDH2 rs671 G>A may be a potential risk factor for AD but not for PD in the East Asians [21]. On the other hand, research by Li et al. published in 2019 showed that PD patients with allele rs671 (A) were more likely to have excessive daytime sleepiness and might tend to have difficulty maintaining sleep. In those authors’ opinion, the results obtained by them suggest that ALDH2 may modulate the accumulation of monoamine neurotransmitters and hence, the non-motor symptoms in patients with PD [22]. Recent studies by Yu et al. have shown the detrimental effect of single nucleotide polymorphism (SNP) of catechol-*o*-methyltransferase (COMT)—COMT rs4680 SNP on the motor symptoms of PD patients, as well as the authors found that ALDH2 rs671 SNP moderated the impact of COMT rs4680 (A) for the symptom of ‘hand movements’ [23]. It should be reminded that the ALDH2 isoenzyme is also involved in removing the products of lipid peroxidation triggered by oxidative stress, like 4-hydroxy-2-nonenal (4-HNE) and malondialdehyde (MDA). It has been shown that 4-HNE concentration increased in the brain tissue of the ALDH2^*^2/2 transgenic mice in an age-dependent manner [24].

Williams et al. found elevated levels of 4-HNE in from post-mortem samples of the hippocampus collected from patients with AD [25].

It is also worth noting that a close relationship between the presence of ALDH2 SNPs and many types of cancer has been reported. It has been shown that ALDH2 SNPs are associated with higher levels of smoking–DNA adducts in blood [26] with higher rates of esophageal cancer [27] and with lung cancer in smokers [28]. It is possibly related to the DNA damaging effects of acetaldehyde [29]. On the other hand, in bladder cancer research, Andrew et al. showed that among people with the ALDH2 rs2238151 genotype variant, alcohol consumption was associated with a longer time to cancer recurrence [30]. It is also known that DSF, an inhibitor of ALDH2, reduces the risk of bladder cancer in rats exposed to nitrosamines [31]. Paradoxically, enhancing the activity of ALDH2 by N-(1,3-benzodioxol-5-ylmethyl)-2,6-dichlorobenzamide, commonly known as Alda-1 inhibited the cancer stemness, proliferation and migration, leading to minimization of DNA damage in lung adenocarcinoma cells [32]. I encourage the readers, who are more interested in the relationship between the activity of ALDH2 and the metabolism of cancer cells, to read a very good paper entitled ‘ALDH2 and Cancer Therapy’ by Wang and Wu. In that paper, the authors have collected many interesting clinical and translational studies on ALDH2 variant and cancer therapy [33].

Thus, ALDH2 is a promising therapeutic target in many diseases. Compounds that either inhibit or activate the catalytic activity of ALDH2 are continuously sought after. The most famous inhibitor of ALDH2 the already mentioned above, DSF was used as early as in the 1940s in the treatment of patients addicted to alcohol (ethanol). The history of ALDH2 activators is much shorter. The above-mentioned compound Alda-1 was the first reported specific activator of ALDH2 [32,34]. The numerous studies have demonstrated that Alda-1 could reduce cardiac ischemic damage, cerebral injury and has the protective effect against the development of neurodegenerative diseases [35]. At the end of this thread, it is worth adding that Alda-1 in humans both activates wildtype ALDH2 and has the ability to restore the inactive forms of ALDH2, i.e. ALDH2*1/2 and ALDH2*2/2 [36]. Similar results were obtained by Hirohashi et al. in mouse studies [37]. Therefore, it is also considered—which may cause a smile and to sound a little funny—to clinically use Alda-1 after alcohol drinking in patients with mutant ALDH2. Therefore, clinical use of Alda-1 after alcohol drinking in patients with mutant ALDH2 is also considered, however paradoxical it may sound.

In turn, ALDH1 enzymes are primarily localized in the cytosol of cells from various tissues. ALDH1 subfamily includes enzymes able to oxidize retinal and aliphatic aldehydes.

They show also high activity for oxidation of aldophosphamide to carboxycyclophosphamide. Aldophosphamide is the metabolic product of cyclophosphamide, which is a commonly used anticancer drug. Cyclophosphamide is one of the most commonly used cytostatics. Thus, high activity of ALDH1 enzymes results in cyclophosphamide resistance in the cancer cells [38].

The ALDH1 subfamily contains three members: aldehyde dehydrogenase 1 family member (ALDH1A1), aldehyde dehydrogenase 2 family member (ALDH1A2) and aldehyde dehydrogenase family member (ALDH1A3). These enzymes use retinal as the main substrate, so they are retinalaldehyde dehydrogenases (EC 1.2.1.36; RALDHs), alternatively known as: RALDH1, RALDH2 and RALDH3, respectively. The general scheme for the reaction catalyzed by these enzymes is shown by the equation:

$$\text{Retinal}+{\text{NAD}}^{+}+{\text{H}}_{2}\text{O}\to \text{Retinoic acid}+\text{NADH}+2{\text{H}}^{+}$$

RA is present in the form of three geometric isomers: atRA, 9-cis RA and 13-cis RA. The RALDHs have a high specificity for the NAD^+^ oxidation of all-trans- or 9-cis-retinal to atRA or 9-cis RA, respectively. Of the three geometric isomers, atRA is the molecule that mediates most biological functions in the body. The form atRA regulates gene expression by activating their cognate nuclear receptors, i.e. retinoic acid receptors (RARs) and retinoid X receptors (RXRs) [39]. Owing to this, atRA exerts potent effects on cell growth, differentiation and apoptosis of normal and neoplastic cells [40]. Thus, atRA is essential in the immunological function, reproduction and embryonic development of the body. In other words, it is a molecule necessary for proper embryonic development and adult body homeostasis [41].

A large part of the current research concerns the role of atRA and RALDHs in cancer development, as well as in cancer prevention and treatment. It has been shown that overexpression of RALDHs correlates with poor prognosis and tumor aggressiveness and is associated with drug resistance in traditional chemotherapy for cancer treatment.

Thus, identification of modulators of several RALDHs/ALDHs may offer new therapeutic options for patients with cancer [42].

The authors of the paper I am discussing here point out that although research related to the search for compounds affecting the activity of RALDHs/ALDHs has been conducted for a long time, however, there is a lack of research on the use of spices and herbs as the sources of naturally occurring modulators of RALDHs/ALDHs activities. To find phytoderived selective modulators of these enzymes, the authors prepared ethanolic extracts of 22 herbs and spices (juniper berry, star anise, mace, shallot, turmeric, chervil, cumin seed, dill seed, dill weed, celery seed, fennel seed, caraway seed, anise seed, marjoram, oregano, rosemary, sage, thyme, cinnamon, laurel, clove and horseradish). The results obtained by the authors showed that most of the tested spice and herb extracts affected the activity of the tested enzymes. Overall, an inhibitory effect (less than 50% activity remained) on the activity of RALDH1, RALDH2 and RALDH3 was shown by 15, 18 and 17 out of 22 of the tested extracts, respectively. The activity of ALDH2 was inhibited by 12 out of 22 tested extracts.

Several extracts showed selective inhibitory effects on two or three out of four tested enzymes. The activity of RALDH1 was most strongly inhibited by the sage and clove extracts (1 and 2.7% activity remained, respectively). Cinnamon and thyme extracts turned out to be the most potent inhibitors of the activity of RALDH2 (0.6 and 4% activity remained, respectively). Next, thyme and star anise extracts inhibited the activity of RALDH3 most strongly (1.2 and 2.7% activity remained, respectively). Interestingly, while in the presence of star anise extract, RALDH3 was almost completely inhibited, RALDH1 was in fact stimulated (133% relative activity) and RALDH2/ALDH2 still remained active at 54.3 and 87.3%, respectively. The authors emphasize that well-known compounds, such as monoterpene trans-anethole (ANE) present in star anise did not inhibit the activity of RALDH3. It suggests that the target compound(s), which may be a potent and selective RALDH3 inhibitor(s), are present in star anise only in trace amounts. It is worth noting that thyme extract also strongly inhibited the activity of ALDH2 (4.34% activity remained). It is impossible in such a short comment to discuss all the results obtained by the authors, so to end this thread I would like to draw your attention to shallot and caraway seed extracts, which very strongly increased the activity of ALDH2 (143.2 and 229.6% relative activity, respectively). The results obtained by the authors are interesting and extremely promising, nevertheless, the problem requires further, in-depth research, not least because in a few cases large dispersion of test results was observed. This is evidenced by the high values of the standard deviation, for example: 229.6 ± 125.1 or 143.2 ± 112.0, which may indicate a lack of statistical significance.

However, I can agree with the authors that their research proposes a promising workflow to find selective modulators for RALDHs and suggests potential sources of selective modulators which are derived from medicinal plants including herbs and spices. Importantly, the results obtained by the authors may not only broaden our knowledge but also provide a basis for the practical applications.

## Abbreviations

AD: Alzheimer’s disease
ALDH: aldehyde dehydrogenase
atRA: all-trans-retinoic acid
COMT: catechol-*o*-methyltransferase
CNS: central nervous system
DA: dopamine
DOPAL: 3,4-dihydroxyphenylacetaldehyde
DOPGAL: 3,4-dihydroxyphenylglycoaldehyde
DSF: disulfiram
EH: essential hypertension
GTN: glyceryl trinitrate
NO: nitric oxide
PD: Parkinson’s disease
RA: retinoic acid
RALDH: retinaldehyde dehydrogenase
SN: substantia nigra
SNP: single nucleotide polymorphism
4-HNE: 4-hydroxy-2-nonenal

## References

[1] Bui T.B.C., Nosaki S., Kokawa M., Xu Y., Kitamura Y. et al. (2021) Evaluation of spice and herb as phyto-derived selective modulators of human retinaldehyde dehydrogenases using a simple in vitro method. Biosci. Rep. 41, BSR20210491, 10.1042/BSR20210491 33950219 PMC8493444

[2] Antille C., Tran C., Sorg O. and Saurat J.H. (2004) Penetration and metabolism of topical retinoids in ex vivo organ-cultured full-thickness human skin explants. Skin Pharmacol. Physiol. 17, 124–128 10.1159/000077238 15087591

[3] Mei X.F., Hu S.D., Liu P.F., Li F., Zhou X.Y., Zhou Y.F. et al. (2020) ALDH2 gene rs671 polymorphism may decrease the risk of essential hypertension. Int. Heart J. 61, 562–570 10.1536/ihj.19-259 32350201

[4] Mittal M., Bhagwati S., Siddiqi M.I. and Naibedya Chattopadhyay N. (2020) A critical assessment of the potential of pharmacological modulation of aldehyde dehydrogenases to treat the diseases of bone loss. Eur. J. Pharmacol. 886, 173541 10.1016/j.ejphar.2020.173541 32896553

[5] Long T.E. (2017) Repurposing thiram and disulfiram as antibacterial agents for multidrug-resistant* Staphylococcus aureus* infections. Antimicrob. Agents Chemother. 61, e00898–e00917 10.1128/AAC.00898-17 28674046 PMC5571293

[6] Kleydman K., Cohen J.L. and Marmur E. (2012) Nitroglycerin: a review of its use in the treatment of vascular occlusion after soft tissue augmentation. Dermatol. Surg. 38, 1889–1897 10.1111/dsu.12001 23205544

[7] Koppaka V., Thompson D.C., Chen Y., Ellermann M., Nicolaou K.C., Juvonen R.O. et al. (2012) Aldehyde dehydrogenase inhibitors: a comprehensive review of the pharmacology, mechanism of action, substrate specificity, and clinical application. Pharmacol. Rev. 64, 520–539 10.1124/pr.111.005538 22544865 PMC3400832

[8] Canuto R.A., Muzio G., Salvo R.A., Maggiora M., Trombetta A., Chantepie J. et al. (2001) The effect of a novel irreversible inhibitor of aldehyde dehydrogenases 1 and 3 on tumour cell growth and death. Chem. Biol. Interact. 130–132, 209–218 10.1016/S0009-2797(00)00280-5 11306045

[9] Cengiz E., Karaca B., Kucukzeybek Y., Gorumlu G., Gul M.K., Erten C. et al. (2010) Overcoming drug resistance in hormone- and drug-refractory prostate cancer cell line, PC-3 by docetaxel and gossypol combination. Mol. Biol. Rep. 37, 1269–1277 10.1007/s11033-009-9501-y 19288219

[10] Orhan I.E. (2016) Potential of natural products of herbal origin as monoamine oxidase inhibitors. Curr. Pharm. Des. 22, 268–276 10.2174/1381612822666151112150612 26561069

[11] Sharma S., Habib S., Sahu D. and Gupta J. (2021) Chemical properties and therapeutic potential of citral, a monoterpene isolated from lemongrass. Med. Chem. 17, 2–12 10.2174/1573406416666191227111106 31880247

[12] Hua Y., Chen H., Zhao X., Liu M., Jin W., Yan W. et al. (2018) Alda-1, an aldehyde dehydrogenase-2 agonist, improves long-term survival in rats with chronic heart failure following myocardial infarction. Mol. Med. Rep. 18, 3159–3166 10.3892/mmr.2018.9309 30066916 PMC6102689

[13] Belmont‐Díaz J.A., Calleja‐Castañeda L.F., Yoval‐Sánchez B. and Rodríguez‐Zavala J.S. (2015) Tamoxifen, an anticancer drug, is an activator of human aldehyde dehydrogenase 1A1. Proteins 83, 105–116 10.1002/prot.24709 25354921

[14] Beretta M., Sottler A., Schmidt K., Mayer B. and Gorren A.C. (2008) Partially irreversible inactivation of mitochondrial aldehyde dehydrogenase by nitroglycerin. J. Biol. Chem. 283, 30735–30744 10.1074/jbc.M804001200 18786921 PMC2576553

[15] Daiber A., Wenzel P., Oelze M. and Münzel T. (2008) New insights into bioactivation of organic nitrates, nitrate tolerance and cross-tolerance. Clin. Res. Cardiol. 97, 12–20 10.1007/s00392-007-0588-7 17938848

[16] Chen Z., Zhang J. and Stamler J.S. (2002) Identification of the enzymatic mechanism of nitroglycerin bioactivation. Proc. Natl. Acad. Sci. U.S.A. 99, 8306–8311 10.1073/pnas.122225199 12048254 PMC123063

[17] Bilska-Wilkosz A., Kotańska M., Górny M., Filipek B. and Iciek M. (2019) Is the mechanism of nitroglycerin tolerance associated with aldehyde dehydrogenase activity? A contribution to the ongoing discussion. Acta Biochim. Pol. 66, 627–632 10.18388/abp.2019_2908 31883320

[18] Goldstein D.S. (2021) The catecholaldehyde hypothesis for the pathogenesis of catecholaminergic neurodegeneration: what we know and what we do not know. Int. J. Mol. Sci. 22, 5999 10.3390/ijms22115999 34206133 PMC8199574

[19] Kamino K., Nagasaka K., Imagawa M., Yamamoto H., Yoneda H., Ueki A. et al. (2000) Deficiency in mitochondrial aldehyde dehydrogenase increases the risk for late-onset Alzheimer’s disease in the Japanese population. Biochem. Biophys. Res. Commun. 273, 192–196 10.1006/bbrc.2000.2923 10873585

[20] Grünblatt E. and Riederer P. (2016) Aldehyde dehydrogenase (ALDH) in Alzheimer’s and Parkinson’s disease. J. Neural Transm. 123, 83–90 10.1007/s00702-014-1320-1 25298080

[21] Chen J., Huang W., Cheng C.H., Zhou L., Jiang G.B. and Hu Y.Y. (2019) Association between *Aldehyde dehydrogenase-2* polymorphisms and risk of Alzheimer’s disease and Parkinson’s disease: a meta-analysis based on 5,315 individuals. Front. Neurol. 10, 290 10.3389/fneur.2019.00290 30984100 PMC6448532

[22] Lin C.Y., Yu R.L., Wu R.M. and Tan C.H. (2019) Effect of ALDH2 on sleep disturbances in patients with Parkinson’s disease. Sci. Rep. 9, 18950 10.1038/s41598-019-55427-w 31831791 PMC6908732

[23] Yu R.L., Tu S.C., Wu R.M., Lu P.A. and Tan C.H. (2021) Interactions of COMT and ALDH2 genetic polymorphisms on symptoms of Parkinson’s disease. Brain Sci. 11, 361 10.3390/brainsci11030361 33808974 PMC8001371

[24] Ohsawa I., Nishimaki K., Murakami Y., Suzuki Y., Ishikawa M. and Ohta S. (2008) Age- dependent neurodegeneration accompanying memory loss in transgenic mice defective in mitochondrial aldehyde dehydrogenase 2 activity. J. Neurosci. 28, 6239–6249 10.1523/JNEUROSCI.4956-07.2008 18550766 PMC6670537

[25] Williams T.I., Lynn B.C., Markesbery W.R. and Lovell M.A. (2006) Increased levels of 4- hydroxynonenal and acrolein, neurotoxic markers of lipid peroxidation, in the brain in Mild Cognitive Impairment and early Alzheimer’s disease. Neurobiol. Aging 27, 1094–1099 10.1016/j.neurobiolaging.2005.06.004 15993986

[26] Wang Y., Ichiba M., Iyadomi M., Zhang J. and Tomokuni K. (1998) Effects of genetic polymorphism of metabolic enzymes, nutrition, and lifestyle factors on DNA adduct formation in lymphocytes. Ind. Health 36, 337–346 10.2486/indhealth.36.337 9810147

[27] Itoga S., Nomura F., Makino Y., Tomonaga T., Shimada H., Ochiai T. et al. (2002) Tandem repeat polymorphism of the CYP2E1 gene: an association study with esophageal cancer and lung cancer. Alcohol Clin. Exp. Res. 26, 15S–19S 10.1111/j.1530-0277.2002.tb02696.x 12198369

[28] Park J.Y., Matsuo K., Suzuki T., Ito H., Hosono S., Kawase T. et al. (2010) Impact of smoking on lung cancer risk is stronger in those with the homozygous aldehyde dehydrogenase 2 null allele in a Japanese population. Carcinogenesis 31, 660–665 10.1093/carcin/bgq021 20093384

[29] Langevin F., Crossan G.P., Rosado I.V., Arends M.J. and Patel K.J. (2011) Fancd2 counteracts the toxic effects of naturally produced aldehydes in mice. Nature 475, 53–58 10.1038/nature10192 21734703

[30] Andrew A.S., Gui J., Hu T., Wyszynski A., Marsit C.J., Kelsey K.T. et al. (2015) Genetic polymorphisms modify bladder cancer recurrence and survival in a USA population-based prognostic study. BJU Int. 115, 238–247 10.1111/bju.12641 24666523 PMC4533837

[31] Frank N., Wiessler M. and Hadjiolov D. (1984) Influence of disulfiram on the metabolism of N-nitrosodiethylamine. IARC Sci. Publ. 57, 519–523 6533043

[32] Li K., Guo W., Li Z., Wang Y., Sun B., Xu D. et al. (2019) ALDH2 repression promotes lung tumor progression via accumulated acetaldehyde and DNA damage. Neoplasia 21, 602–614 10.1016/j.neo.2019.03.008 31071657 PMC6506700

[33] Wang L.S. and Wu Z.X. (2019) ALDH2 and cancer therapy. Adv. Exp. Med. Biol. 1193, 221–228 10.1007/978-981-13-6260-6_13 31368107

[34] Chen C.H., Ferreira J.C., Gross E.R. and Mochly-Rosen D. (2014) Targeting aldehyde dehydrogenase 2: new therapeutic opportunities. Physiol. Rev. 94, 1–34 10.1152/physrev.00017.2013 24382882 PMC3929114

[35] Kimura M., Yokoyama A. and Higuchi S. (2019) Aldehyde dehydrogenase-2 as a therapeutic target. Expert Opin. Ther. Targets 23, 955–966 10.1080/14728222.2019.1690454 31697578

[36] Perez-Miller S., Younus H., Vanam R., Chen C.H., Mochly-Rosen D. and Hurley T.D. (2010) Alda-1 is an agonist and chemical chaperone for the common human aldehyde dehydrogenase 2 variant. Nat. Struct. Mol. Biol. 17, 159–164 10.1038/nsmb.1737 20062057 PMC2857674

[37] Hirohashi K., Ohashi S., Amanuma Y., Nakai Y., Ida T., Baba K. et al. (2020) Protective effects of Alda-1, an ALDH2 activator, on alcohol-derived DNA damage in the esophagus of human ALDH2*2 (Glu504Lys) knock-in mice. Carcinogenesis 41, 194–202 10.1093/carcin/bgz091 31074772 PMC7175241

[38] Sloderbach A., Górska A., Sikorska M., Misiura K. and Hładoń B. (2013) Classical oxazaphosphorines–metabolism and therapeutic properties–new implications. Postepy. Hig. Med. Dosw. 67, 1235–1253, Polish 10.5604/17322693.1079389 24379264

[39] Mangelsdorf D.J. (1994) Vitamin A receptors. Nutr. Rev. 52, S32–S44 10.1111/j.1753-4887.1994.tb01385.x 8202281

[40] Hoffman C. and Eichele G. (1994) Retinoids in development. In The Retinoids: Biology, Chemistry and Medicine, 2nd edn, (Sporn M.B., Roberts A.B. and Goodman D.S., eds), pp. 387–442, Raven Press, New York

[41] Kam R.K., Deng Y., Chen Y. and Zhao H. (2012) Retinoic acid synthesis and functions in early embryonic development. Cell Biosci. 2, 11 10.1186/2045-3701-2-11 22439772 PMC3325842

[42] Harper A.R., Le A.T., Mather T., Burgett A., Berry W. and Summers J.A. (2018) Design, synthesis, and ex vivo evaluation of a selective inhibitor for retinaldehyde dehydrogenase enzymes. Bioorg. Med. Chem. 26, 5766–5779 10.1016/j.bmc.2018.10.009 30409702 PMC6331054

